# Supplementary material for: Indian Ocean glacial deoxygenation and respired carbon accumulation during mid-late Quaternary ice ages
Source: Nat Commun. 2023 Aug 10;14:4841. doi: 10.1038/s41467-023-40452-1 (PMC10415292; doi:10.1038/s41467-023-40452-1)
Supplement: Supplementary file 1 — Supplementary Information [file 41467_2023_40452_MOESM1_ESM.pdf]

Supplementary Information for

Indian Ocean glacial deoxygenation and respired carbon accumulation during mid-late Quaternary ice ages

Liao Chang[1,2*], Babette A. A. Hoogakker[3], David Heslop[4], Xiang Zhao[4], Andrew P. Roberts[4], Patrick De Deckker[4], Pengfei Xue[1], Zhaowen Pei[1], Fan Zeng[1], Rong Huang[1], Baoqi Huang[1], Shishun Wang[1], Thomas A. Berndt[5], Melanie Leng[6,7], Jan-Berend W. Stuut[8], Richard J. Harrison[9]

[1] Laboratory of Orogenic Belts and Crustal Evolution, School of Earth and Space Sciences, Peking University, 100871 Beijing, China

[2] Laboratory for Marine Geology, Qingdao National Laboratory for Marine Science and Technology, 266071 Qingdao, China

[3] The Lyell Centre, Heriot-Watt University, Edinburgh, EH14 4BA, UK

[4] Research School of Earth Sciences, The Australian National University, Canberra, ACT 2601, Australia

[5] Department of Geophysics, School of Earth and Space Sciences, Peking University, 100871 Beijing, China

[6] National Environmental Isotope Facility, British Geological Survey, Keyworth, NG12 5GG, UK

[7] School of Biosciences, University of Nottingham, Sutton Bonington, LE12 5RD, UK

[8] NIOZ-Royal Netherlands Institute for Sea Research and Utrecht University, Texel, The Netherlands

[9] Department of Earth Sciences, University of Cambridge, Cambridge, CB2 3EQ, UK

Correspondence to: Liao Chang
Email: liao.chang@pku.edu.cn

This PDF file includes:

Supplementary Texts S1 to S3
Supplementary Figures S1 to S7
Supplementary Table S1 to S3
SI References

Supplementary Information Text

Text S1. Marine sediment proxies for tracing bottom-water oxygenation

The rationale behind use of the main marine sediment proxies for reconstructing oceanic bottom-water oxygenation is outlined briefly here. A succinct overview of several proxies is given by Moffitt et al.[1].

(i) Sediment laminations. Oxygen deficiency excludes various benthic species and, therefore, prevents bioturbation. This results in preservation of sediment laminations. For example, van Geen et al.[2] studied sediment laminations in the northeast Pacific Ocean during the last deglaciation and Nicolo et al.[3] analysed bioturbation images of

sediments to trace South Pacific Ocean oxygen levels across the Paleocene-Eocene thermal maximum (PETM).

(ii) Foraminiferal assemblages. Benthic foraminiferal assemblages and abundances are influenced by dissolved oxygen concentration and other conditions, such as organic matter flux[4]. Therefore, properties of benthic foraminiferal assemblages can be used to trace bottom-water oxygen concentration (e.g., in analysing sedimentary PETM records[5]). Recently, Rathburn et al.[6] proposed a paleo-oxygen proxy based on foraminiferal morphology.

(iii) Redox sensitive trace metals. Under more reducing conditions, some redox-sensitive metals, including Mn, Mo, Cd, and U, diffuse from the water column across the sediment-water interface and are enriched in reducing sedimentary intervals. Choumiline et al. provide a modern overview of redox proxies[7]. Chun et al.[8] applied trace metal analysis to PETM sediments to infer bottom-ocean oxygenation changes. Trace element enrichment factors can be used to trace the paleoredox environment of sediments at or near the time of deposition. These metals are incorporated within subsurface sediments, which reflect the redox state of pore waters rather than the overlying bottom waters.

(iv) Benthic foraminiferal carbon-isotope gradients ($\Delta\delta^{13}C$). The $\delta^{13}C$ difference between bottom waters and pore waters across the oxic to anoxic boundary in sediments ($\Delta\delta^{13}C$) is related to the oxygen concentration of the overlying bottom water[9]. $\delta^{13}C$ signals of benthic foraminifera from microhabitats in bottom waters (*Cibicidoides wuellerstorfi*) and sediments across the oxic to anoxic boundary (*Globobulimina spp.*) have been measured to give the $\Delta\delta^{13}C$ between bottom water and pore water at the anoxic boundary[10]. The benthic foraminiferal $\Delta\delta^{13}C$ method provides a quantitative approach for tracing bottom-water oxygenation, and has been used to study recent glacial-interglacial oxygenation changes[10,11]. This method is limited to environments where high organic carbon fluxes facilitate development of a sedimentary anoxic boundary. Also, at high sediment accumulation sites where oxygen penetration exceeds light carbon accumulation in the sediment mixed layer, sulfate reduction and denitrification can complicate the relationship between *C. wuellerstorfi* and *Globobulimina* spp.[9,12].

In addition to sedimentological, paleontological, and geochemical tracers, various sedimentary magnetic parameters have been suggested as tracers of variable bottom water oxygenation. Some sediment magnetic properties are sensitive to bottom-water oxygenation conditions that reflect magnetic mineral diagenesis associated with redox-sensitive conditions[13]. Signatures of magnetic mineral diagenesis have been applied to study sapropel-bearing Eastern Mediterranean sediments[14], organic-rich hemipelagic Pacific Ocean sediments[15], and carbonate-

free abyssal Pacific Ocean sediments[16]. Magnetofossil signatures have also been used to study glacial-interglacial cycles[17–19], Eocene and older pelagic sediments[20,21], and the PETM[22] to trace deep-sea oxygenation conditions.

Text S2. Additional rock magnetic and geochemical properties of the studied core

We present here additional rock magnetic data and analyses for typical marine sediment samples from interglacial and glacial intervals above 17.55 m in the studied core MD00-2361, including first-order reversal curve (FORC) diagrams (Supplementary Figure S1) measured with a vibrating sample magnetometer (VSM), fitting of alternating field (AF) demagnetization of saturation isothermal remanent magnetization (SIRM) data (Supplementary Figure S2), and fitting of AF demagnetization of ARM data (Supplementary Figure S3) from cryogenic magnetometer measurements. Coercivity enhancement for the glacial samples compared to interglacial samples can be clearly seen. ARM fitting results (Supplementary Figure S3) have similar down-core trends as for IRM curve fitting. The main difference is a suppression of the lowest field component compared to more pronounced BS and BH components in the ARM data (Supplementary Figure S3) because ARM is more sensitive to fine magnetic particles, including magnetofossils.

Down-core magnetic and X-ray fluorescence (XRF) elemental variations indicate a magnetization decrease at ~17.55 m, likely due to magnetic mineral dissolution[13]. Below this depth, magnetofossil populations are dissolved (Supplementary Figure S4).This abrupt magnetization decrease is consistent with magnetic property changes. For example, samples below the sulfate-reduction redox boundary (i.e., below ~17.55 m) do not have the central ridge FORC signature associated with biogenic magnetite or biogenic greigite, but instead have FORC characteristics typical of interacting single-domain/vortex state assemblages with broad vertical spread[21–23] (Supplementary Figure S1j). These data support the interpretations in the main text.

Text S3. Micromagnetic simulations of magnetofossil assemblages

To understand the link between magnetofossil ensembles and magnetic properties, we made three-dimensional micromagnetic simulations[24] using magnetofossil size and shape data from TEM observations for glacial and interglacial samples (Figure 3, main paper). Micromagnetic simulation of FORC diagrams and coercivity distributions for two typical samples are presented in Supplementary Figure S6. Simulated magnetic parameters are listed in Supplementary Table S1. Exact chain configurations within the studied sediment samples are unknown, so various chain architectures were modeled, with variable number (n) and separation (d) of particles in a chain, chain bending (c), and chain packing factors[22–24]. Simulations indicate that all of these factors can affect the magnetic properties of magnetofossil assemblages (Supplementary Table S1, Figure S6), but the same trend as that obtained from experimental magnetic properties (i.e., magnetofossil coercivity increases from interglacial to glacial samples) is predicted when using

the same model parameters for different magnetofossil assemblages. For straight chains with a small particle gap (*d* = 5 nm), predicted coercivity values are close to the saturation value, so individual magnetofossil crystal size is not important (Supplementary Table S1). For fully collapsed chains with a larger particle gap, the size-dependent coercivity is significant. The calculated coercivity hardening from interglacial to glacial samples (Supplementary Figure S6) follows the experimental data trend (Figure 2; Supplementary Figures S1-S3).

In summary, micromagnetic simulations confirm the same trend between experimental magnetic properties (Figure 2 of the main paper) and magnetofossil size distributions (Figure 3, main paper), i.e., magnetofossil coercivity and crystal size/elongation increase from interglacial to glacial samples. Our simulations validate directly the use of magnetic coercivity distributions (i.e., δ_{BH}) for characterizing magnetofossil morphology.

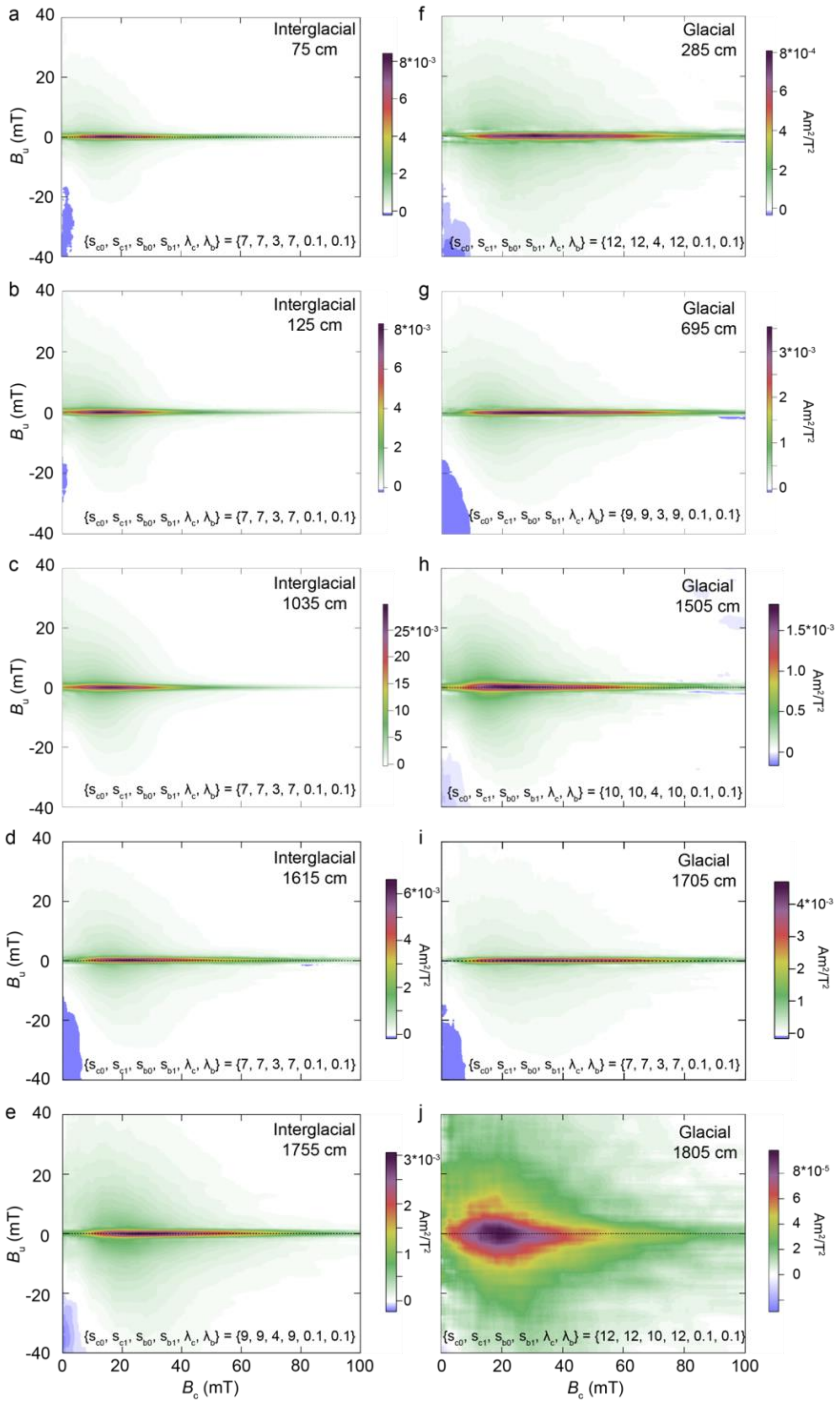

Figure S1. First-order reversal curve (FORC) diagrams for typical marine sediment samples from (a–e) interglacial and (f–i) glacial stages, and (j) a sample below 17.55 m within the sulphate reducing diagenetic interval in core MD00-2361. FORC diagrams were processed using FORCinel version 3.06[25] with the VARIFORC protocol[26]. Smoothing parameters are indicated at the base of each plot.

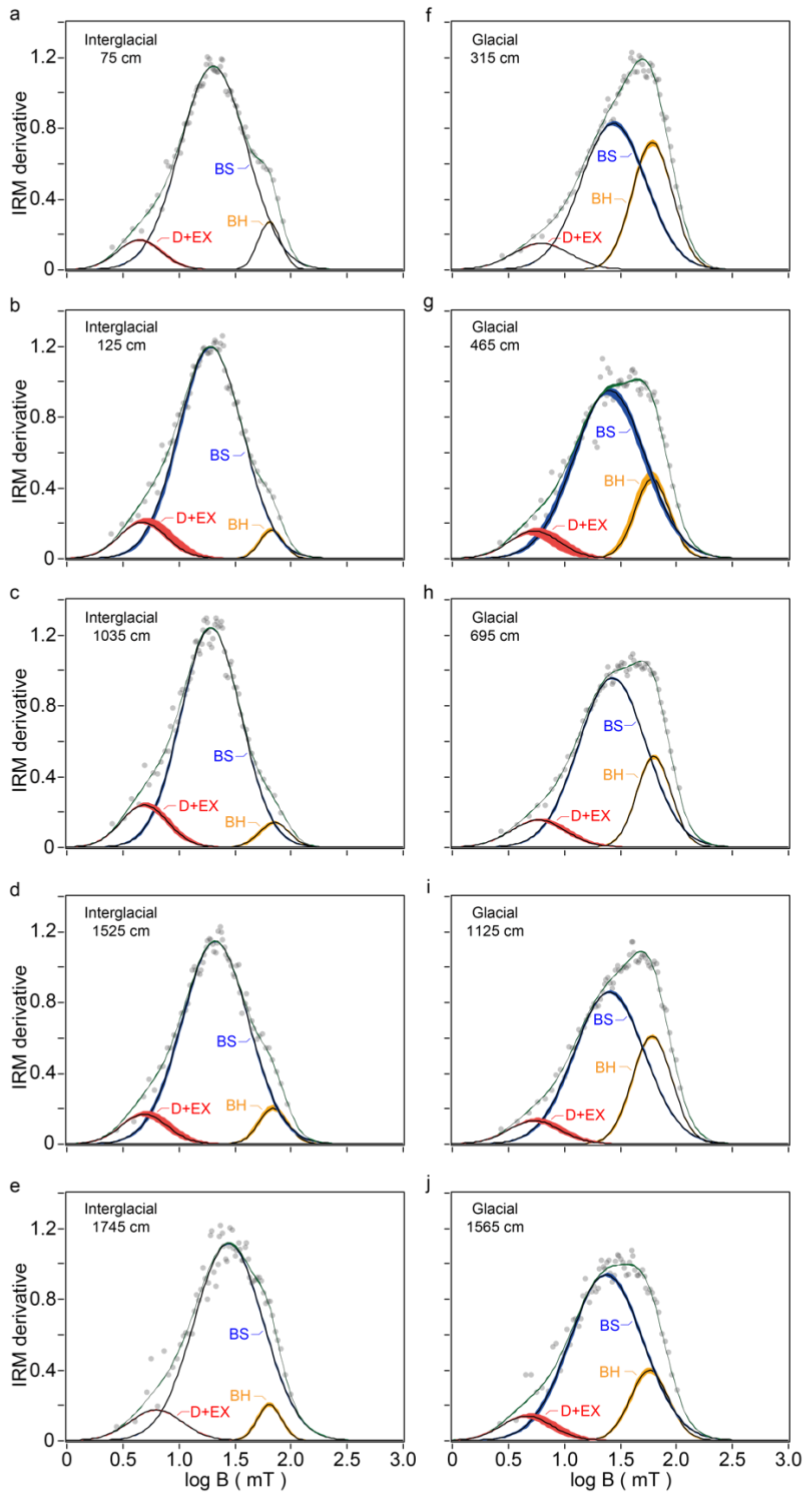

Figure S2. Fitting (green) of alternating field (AF) demagnetization of isothermal remanent magnetization (IRM) data (open circles) with three log-Gaussian components (red, blue, and orange) for typical (a–e) interglacial and (f–j) glacial samples. Shaded colored areas represent 95% confidence intervals calculated based on a resampling routine.

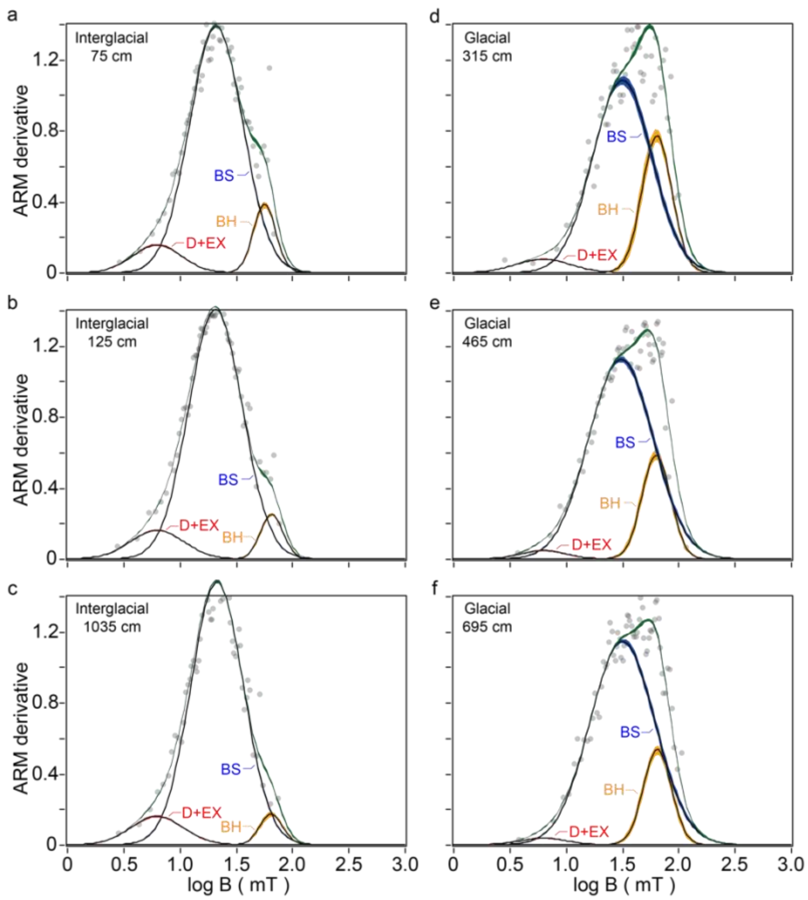

Figure S3. Fitting (green) of alternating field (AF) demagnetization of anhysteretic remanent magnetization (ARM) data (open circles) with three log-Gaussian components (red, blue, and orange) for typical (a–c) interglacial and (d–f) glacial samples. Shaded colored areas represent error envelopes of 95% confidence intervals calculated based on a resampling routine.

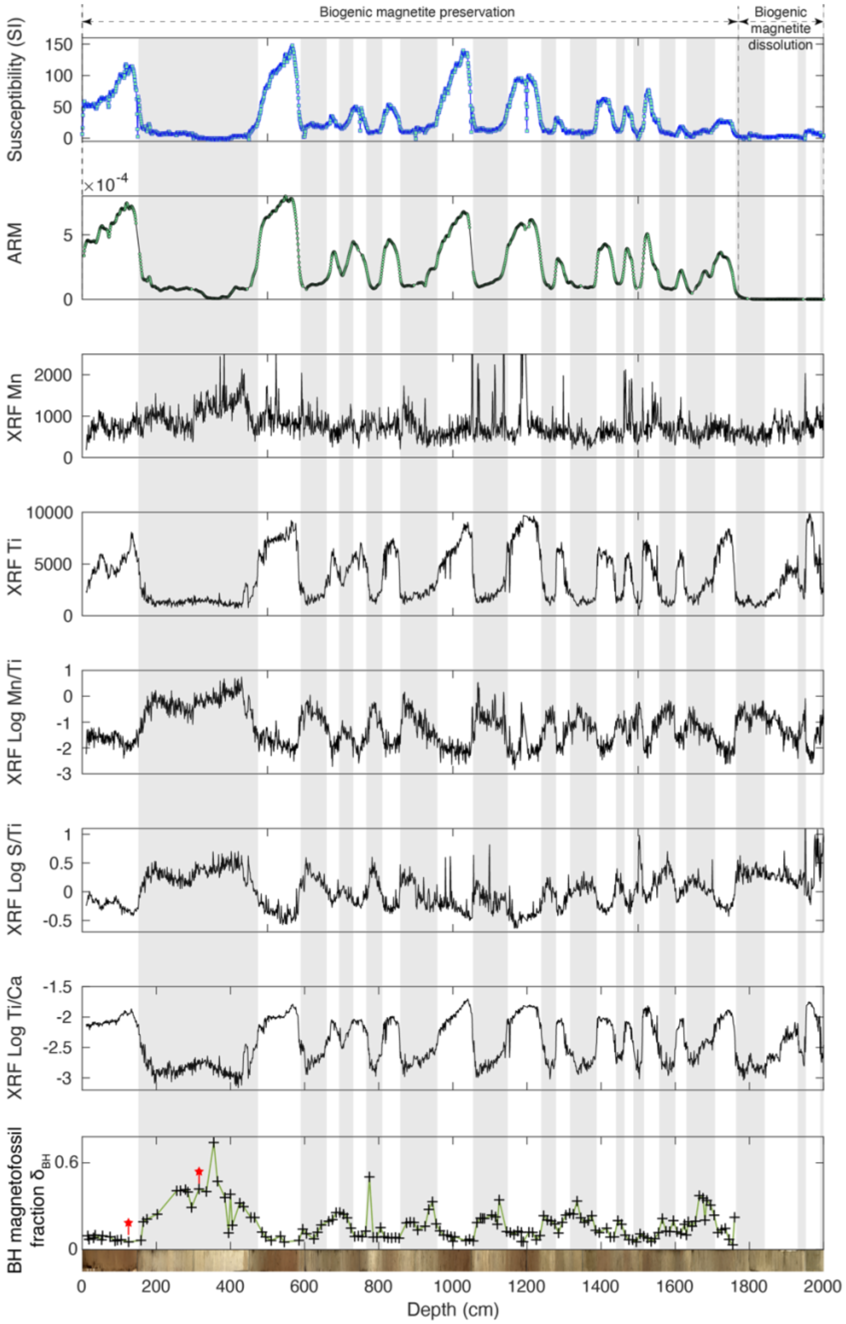

Figure S4. Down-core depth profiles for the uppermost 20 m of core MD00-2361. Magnetic susceptibility and anhysteretic remanent magnetization (ARM) intensity are from u-channel measurements. Grey bars mark intervals with lower magnetization and X-ray fluorescence (XRF) Ti/Ca values for core MD00-2361, which broadly correspond to glacial periods. Core images at the bottom of the figure indicate sediment color variations. Red stars indicate the stratigraphic position of two sediment samples selected for transmission electron microscope (TEM) observations of magnetofossils (Figure 3, main paper).

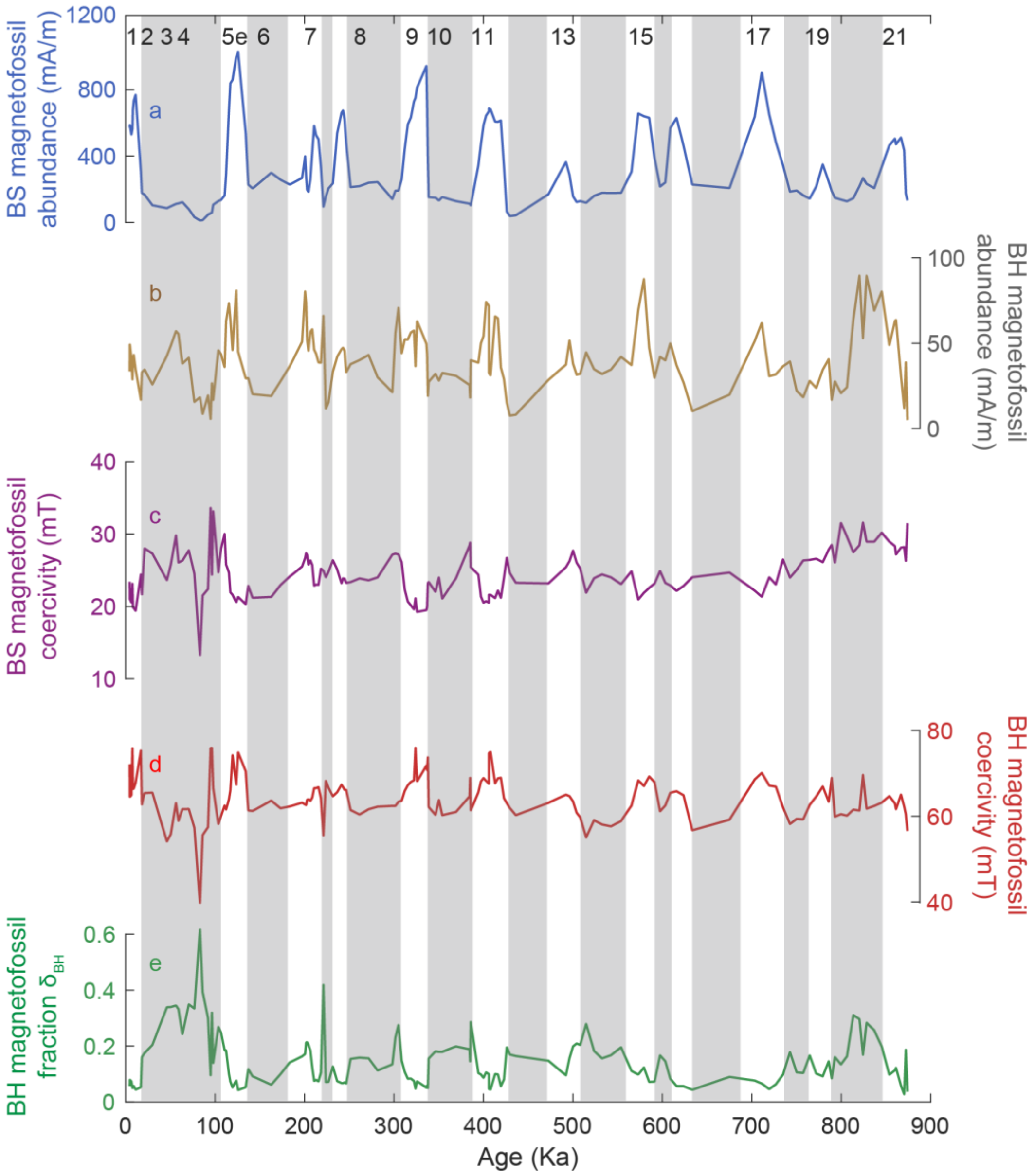

Figure S5. Down-core depth profiles of biogenic soft (BS) and biogenic hard (BH) magnetofossil populations for the uppermost 20 m of core MD00-2361. (a, b) Absolute abundance of BS and BH magnetofossils, respectively, and (c, d) corresponding coercivity changes. (e) BH magnetofossil fraction δ_{BH}.

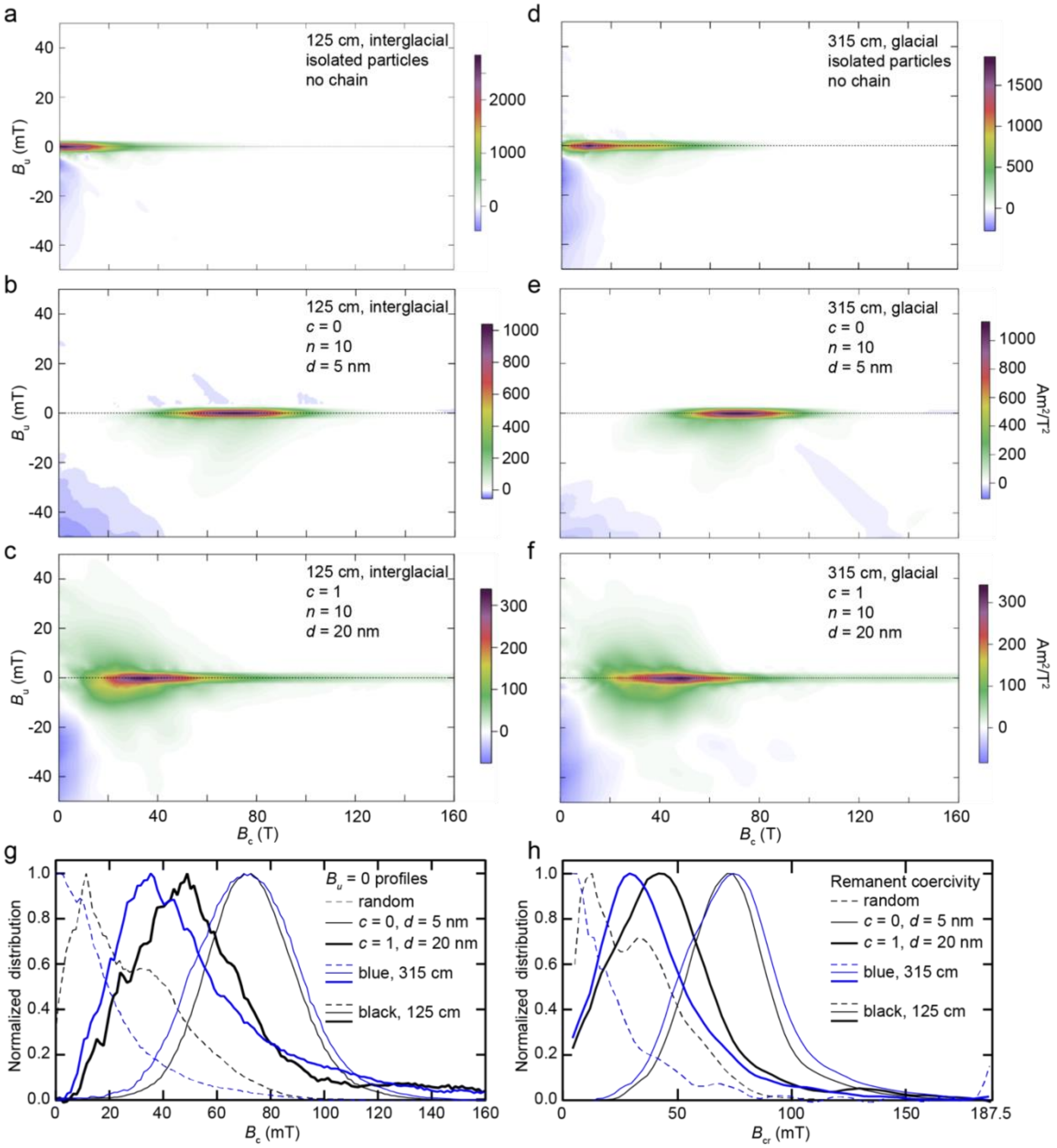

Figure S6. Micromagnetic simulations of the magnetic properties of two typical samples (interglacial sample at 125 cm, glacial sample at 315 cm) using magnetofossil morphological data from transmission electron microscope (TEM) observations (Fig. 3 of the main paper). (a–f) Examples of simulated first-order reversal curve (FORC) diagrams and (g) extracted coercivity profiles along $B_u = 0$. (h) Extracted coercivity of remanence profiles. Different microstructures of magnetofossil assemblages were modeled: *n*: particles in a chain (*n* = 10); *d*: particle gap in a chain; *c*: chain bending factor (*c* = 0 represents a straight chain, *c* = 1 represents fully collapsed chains). FORC diagrams were processed using FORCinel[25] with VARIFORC smoothing parameters[26]: $\{s_{c0}, s_{c1}, s_{b0}, s_{b1}, \lambda_c, \lambda_b\} = \{7, 7, 3, 7, 0.1, 0.1\}$.

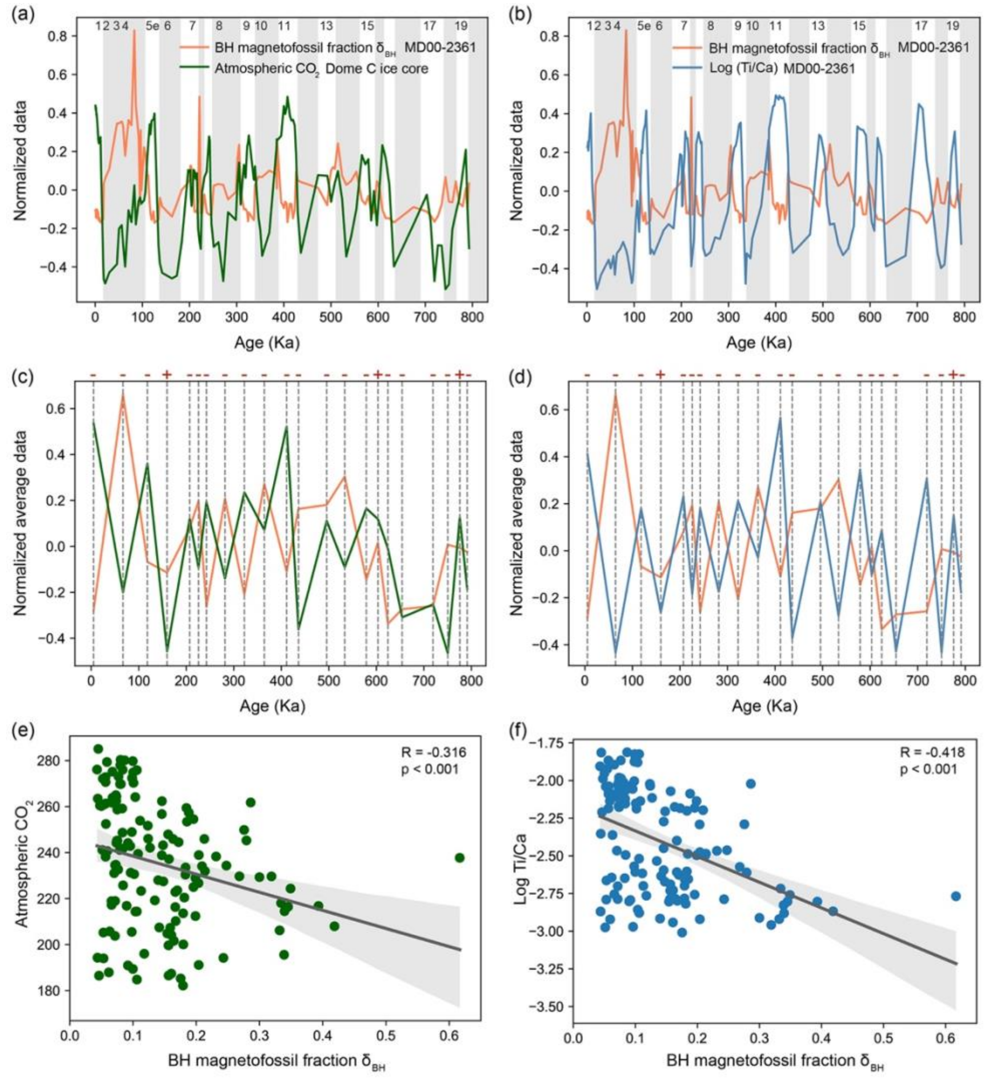

Figure S7. (a, b) Min-max normalization, (c, d) normalized average data for each glacial and interglacial interval, and (e, f) linear regression analysis of biogenic hard (BH) magnetofossil fraction δ_{BH} in core MD00-2361 with atmospheric CO_2 variation recorded in the Dome C ice core[27] and X-ray fluorescence (XRF) Ti/Ca ratio in core MD00-2361. In (a, b), grey bars mark intervals with lower planktonic $\delta^{18}O$ and XRF Ti/Ca values for core MD00-2361, which broadly correspond to glacial periods. In (c, d), the dashed lines indicate the mid-point of each glacial and interglacial interval. "-" and "+" signs correspond to inverse and positive correlations, respectively. In (e, f), the Spearman correlation coefficient *R* obtained by regression and the p-value of the t-test of regression coefficients are included. Black solid lines indicate trend defined by a linear function with 95% confidence interval (grey area).

Table S1. Simulated and experimental hysteresis parameters for two typical samples (interglacial 125-cm core depth and glacial 315-cm core depth) from the studied core MD00-2361.

Sample and depth in the core	Bulk/modeled parameters			Hysteresis parameters			
	*n**	*d* (nm)*	*c**	B_c (mT)	B_{cr} (mT)	M_{rs}/M_s	B_{cr}/B_c
MD00-2361-125	experimental data			10.4	23.8	0.255	2.291
MD00-2361-125	10	5	0	53.8	73.8	0.492	1.373
MD00-2361-125	10	20	1	21.2	38.8	0.340	1.827
MD00-2361-125#	1	∞	1	7.1	15.1	0.498	2.139
MD00-2361-315	experimental data			27.5	49.3	0.388	1.794
MD00-2361-315	10	5	0	54.7	72.4	0.497	1.323
MD00-2361-315	10	20	1	26.8	45.7	0.380	1.706
MD00-2361-315#	1	∞	1	14.0	28.1	0.502	2.007

**n* = number of particles in a chain; **d* = particle gap in a chain; **c* = degree of chain bending;
randomly oriented particles without chains.

Table S2. $\delta^{18}O$, $\delta^{13}C$, $\Delta\delta^{13}C$ of benthic foraminifera *C. wuellerstorfi* and *Globobulimina spp.* for four samples from core MD00-2361 and reconstructed bottom-water oxygenation concentration.

Depth (cm)	Age (kyr BP)	$\delta^{18}O$ *C. wueller storfi*	$\delta^{13}C$ *C. wueller storfi*	$\delta^{18}O$ *Globobulimina spp.*	$\delta^{13}C$ *Globobulimina spp.*	$\Delta\delta^{13}C$	$[O_2]$ (μmol/kg)
280	53.4	3.10	0.46	4.19	-0.4	0.86	58
1100	342.3	3.07	0.34	3.91	-0.97	1.31	115
1233	423.3	2.23	0.61	3.34	0.61	1.57	150
1630	788.8	3.22	0.52	3.99	-0.85	1.37	123

Table S3. Authigenic uranium (aU), redox-sensitive trace metal concentrations and their normalised values for selected samples from core MD00-2361.

Depth (cm)	Age (ka)	Al (wt%)	Cd (ppm)	Mo (ppm)	U (ppm)	Th (ppm)	aU (ppm)	U/Al (ppm/wt%)	Cd/Al (ppm/wt%)	Mo/Al (ppm/wt%)
365	86.09	0.662	0.422	0.372	5.708	1.655	5.046	8.623	0.638	0.562
400	96.45	0.499	0.468	0.568	4.911	1.102	4.470	9.850	0.938	1.139
510	119.53	3.675	0.646	0.624	5.176	6.746	2.478	1.408	0.176	0.170
545	125.71	4.170	0.548	0.582	5.026	7.725	1.937	1.205	0.131	0.140
1000	324.09	4.350	0.598	0.540	5.051	7.926	1.881	1.161	0.138	0.124
1045	336.99	4.317	0.766	1.215	4.600	7.235	1.706	1.065	0.177	0.281
1055	337.87	1.585	0.492	0.386	4.983	2.734	3.889	3.145	0.310	0.243
1080	350.17	0.804	0.504	0.244	4.578	1.480	3.986	5.693	0.627	0.303
1120	385.10	1.187	0.220	0.290	3.948	2.189	3.072	3.325	0.185	0.244
1155	397.20	4.256	0.749	0.482	6.515	7.592	3.478	1.531	0.176	0.113
1175	402.99	4.764	0.891	0.598	5.966	8.409	2.602	1.252	0.187	0.126
1185	406.14	4.718	0.572	0.658	6.601	8.165	3.335	1.399	0.121	0.139
1190	407.85	5.183	0.746	0.734	6.311	9.159	2.647	1.218	0.144	0.142
1255	429.46	0.967	0.606	0.314	6.071	1.961	5.287	6.279	0.627	0.325
1265	436.19	1.031	0.402	0.220	5.859	2.136	5.004	5.681	0.390	0.213
1625	786.01	2.458	0.470	0.244	6.055	4.629	4.203	2.464	0.191	0.099
1630	789.43	1.167	0.466	0.200	6.186	2.249	5.287	5.301	0.399	0.171
1680	824.31	2.076	0.596	0.306	7.777	4.137	6.122	3.746	0.287	0.147
1745	866.80	4.268	0.576	0.362	4.790	7.742	1.694	1.122	0.135	0.085
1760	872.22	1.582	0.418	0.250	4.498	3.563	3.072	2.843	0.264	0.158

Supplementary References

1. S. E. Moffitt, R. A. Moffitt, W. Sauthoff, C. V. Davis, K. Hewett, T. M. Hill, Paleoceanographic insights on recent oxygen minimum zone expansion: Lessons for modern oceanography. *PLoS ONE* **10**, e0115246 (2015).
2. A. Van Geen, R. G. Fairbanks, P. Dartnell, M. McGann, J. V. Gardner, M. Kashgarian, Ventilation changes in the northeast Pacific during the last deglaciation. *Paleoceanography* **11**, 519–528 (1996).
3. M. J. Nicolo, G. R. Dickens, C. J. Hollis, South Pacific intermediate water oxygen depletion at the onset of the Paleocene-Eocene thermal maximum as depicted in New Zealand margin sections. *Paleoceanography* **25**, PA4210 (2010).
4. B. Corliss, Microhabitat of benthic foraminifera within deep-sea sediments. *Nature* **314**, 435–438 (1985).
5. E. Thomas, "Biogeography of the Late Paleocene benthic foraminiferal extinction" in Late Paleocene–Early Eocene biotic and climatic events in the marine and terrestrial records, M.-P. Aubry, S. G. Lucas, W. A. Berggren, Eds. (Columbia University Press, New York, 1998), pp. 214–243.
6. E. Rathburn, J. Willingham, W. Ziebis, A. M. Burkett, B. H. Corliss, A new biological proxy for deep-sea paleo-oxygen: Pores of epifaunal benthic foraminifera. *Sci. Repts* **8**, 1–8 (2018).
7. K. Choumiline, L. Pérez-Cruz, A. B. Gray, S. M. Bates, T. W. Lyons, Scenarios of deoxygenation of the Eastern Tropical North Pacific during the past millennium as a window into the future of oxygen minimum zones. *Front. Earth Sci.* **7**, 237 (2019).
8. C. O. J. Chun, M. L. Delaney, J. C. Zachos, Paleoredox changes across the Paleocene–Eocene thermal maximum, Walvis Ridge (ODP Sites 1262, 1263, and 1266): Evidence from Mn and U enrichment factors. *Paleoceanography* **25**, PA4202 (2010).
9. D. C. McCorkle, S. R. Emerson, The relationship between pore water carbon isotopic composition and bottom water oxygen concentration. *Geochim. Cosmochim. Acta* **52**, 1169–1178 (1988).
10. B. A. A. Hoogakker, H. Elderfield, G. Schmiedl, I. N. McCave, R. E. M. Rickaby, Glacial-interglacial changes in bottom-water oxygen content on the Portuguese margin. *Nat. Geosci.* **8,** 40–43 (2015).
11. B. A. A. Hoogakker, Z. Lu et al., Glacial expansion of oxygen-depleted seawater in the eastern tropical Pacific. *Nature* **562,** 410–413 (2018).
12. S. L. Jaccard, E. D. Galbraith, Large climate-driven changes of oceanic oxygen concentrations during the last deglaciation. *Nat. Geosci.* **5,** 151–156 (2012).
13. A. P. Roberts, Magnetic mineral diagenesis. *Earth-Sci. Rev.* **151,** 1–47 (2015).
14. J. C. Larrasoaña, A. P. Roberts, J. S. Stoner, C. Richter, R. Wehausen, A new proxy for bottom-water ventilation in the eastern Mediterranean based on diagenetically controlled magnetic properties of sapropel-bearing sediments. *Palaeogeogr. Palaeoclimatol. Palaeoecol.* **190**, 221–242 (2003).
15. L. Chang, et al., Asian monsoon modulation of nonsteady state diagenesis in hemipelagic marine sediments offshore of Japan. *Geochem. Geophys. Geosyst.* **17**, 4383–4398 (2016).
16. L. Korff, T. von Dobeneck, T. Frederichs, S. Kasten, G. Kuhn, R. Gersonde, B. Diekmann, Cyclic magnetite dissolution in Pleistocene sediments of the abyssal northwest Pacific Ocean: Evidence for glacial oxygen depletion and carbon trapping. *Paleoceanography* **31**, 600–624, (2016).
17. P. P. Hesse, Evidence for bacterial palaeoecological origin of magnetic mineral cycles in oxic and sub-oxic Tasman Sea sediments. *Mar. Geol.* **117,** 1–17 (1994).
18. T. Yamazaki, M. Ikehara, Origin of magnetic mineral concentration variation in the Southern Ocean. *Paleoceanography* **27,** PA2206 (2012).
19. T. Yamazaki, Paleoposition of Intertropical Convergence Zone in the eastern Pacific inferred from glacial-interglacial changes in terrigenous and biogenic magnetic mineral fractions. *Geology* **40,** 151–154 (2012).
20. L. Chang et al., Low-temperature magnetic properties of pelagic carbonates: Oxidation of biogenic magnetite and identification of magnetosome chains. *J. Geophys. Res. Solid Earth* **118**, 6049–6065 (2013).

21. T. Yamazaki, T. Shimono, Abundant bacterial magnetite occurrence in oxic red clay. *Geology* **41,** 1191–1194 (2013).
22. L. Chang et al., Coupled microbial bloom and oxygenation decline recorded by magnetofossils during the Palaeocene-Eocene Thermal Maximum. *Nat. Commun.* **9**, 4007 (2018).
23. R. J. Harrison, I. Lascu, FORCulator: A micromagnetic tool for simulating first-order reversal curve diagrams. *Geochem. Geophys. Geosyst.* **15,** 4671–4691 (2014).
24. L. Chang, R. J. Harrison, T. A. Berndt, Micromagnetic simulation of magnetofossils with realistic size and shape distributions: Linking magnetic proxies with nanoscale observations and implications for magnetofossil identification. *Earth Planet. Sci. Lett.* **527**, 115790 (2019).
25. R. J. Harrison, J. M. Feinberg, FORCinel: An improved algorithm for calculating first-order reversal curve distributions using locally weighted regression smoothing. *Geochem. Geophys. Geosyst.* **9,** Q05016 (2008).
26. R. Egli, VARIFORC: An optimized protocol for calculating non-regular first-order reversal curve (FORC) diagrams. *Glob. Planet. Change* **110,** 302–320 (2013).
27. D. Luthi, et al., High-resolution carbon dioxide concentration record 650,000–800,000 years before present. *Nature* **453**, 379–382 (2008).
